# Winter activity and virus infection of *Culicoides* biting midges (Diptera: Ceratopogonidae) in German livestock housing before and during the 2024 BTV-3 outbreak

**DOI:** 10.1186/s13071-026-07620-w

**Published:** 2026-09-04

**Authors:** Anja Voigt, Helge Kampen, Martin Beer, Kerstin Wernike, Dorothee Scheuch, Franziska Sick, Sophie Zeiske, Doreen Werner

**Affiliations:** 1 Leibniz-Centre for Agricultural Landscape Research, Muencheberg, Germany; 2 Friedrich-Loeffler-Institut, Federal Research Institute for Animal Health, Greifswald - Insel Riems, Germany

**Keywords:** *Culicoides*, Germany, Livestock, Population surveillance, Vector, Seasonal variation

## Abstract

**Background:**

Despite its importance for *Culicoides*-borne disease risk assessment, the winter activity of species of this biting midge genus remains poorly characterized. Moreover, information on virus circulation in winter-active *Culicoides* vectors, both under endemic conditions and during outbreaks, is almost non-existent.

**Methods:**

UV-light traps to collect *Culicoides* biting midges were operated once a week for 24 h in cattle, sheep, goat and horse stables at 12 to 55 locations during four consecutive winter periods (November 1 to March 31, 2021 to 2025) throughout Germany. Collected females of the genus *Culicoides* were morphologically assigned to Obsoletus Group or Pulicaris Complex. Temperature data were recorded to relate biting midge activity to temperatures. Screening for bluetongue virus (BTV), Schmallenberg virus and epizootic hemorrhagic disease virus was done by real-time RT-PCR on 1,581 pools (46,358 females) of both groups collected before (winter 2022/2023) and during the BTV serotype 3 (BTV-3) outbreak (winters 2023/2024 and 2024/2025).

**Results:**

*Culicoides* collections displayed notable variation between sites and months. November and March accounted for 95% of captures, while mid-winter months (December to February) yielded only 5%, with January and February each contributing 1% of total captures. The March 2024 and 2025 activity peaks coincided with mild temperatures and the inclusion of new monitoring sites, suggesting that one or both factors contributed. Although blood-fed females were very rare during mid-winter, their abundance often exceeded the EU threshold for parous *Culicoides* to define a vector-free period of < 5 individuals per trap and night. However, only a single *Culicoides* pool from November 2024 tested positive for BTV-3-RNA. All other pools were negative for all three viruses.

**Conclusions:**

Potential *Culicoides* vectors are active and feed on blood in livestock housings during wintertime. The presence of blood-fed females, exceeding the EU threshold, challenges the concept of a ‘vector-free period’. However, despite extensive monitoring over four years including outbreak conditions, only one virus-positive *Culicoides* pool was detected during winter. Combined with the very low number of biting midges and the temperatures not allowing virus replication in arthropods, virus circulation during wintertime appears to be hardly existent even under endemic or outbreak conditions. Considering both *Culicoides* activity and virus circulation, the findings suggest that the risk of BTV infection in ruminants via biting midges during the central European winter (December to March) is negligible. BTV overwintering in central Europe likely depends on either rare infection events in ruminants and vectors during winter that sustain viremia until spring, or on persistence mechanisms that are currently unknown.

**Graphical Abstract:**

**Figure:**
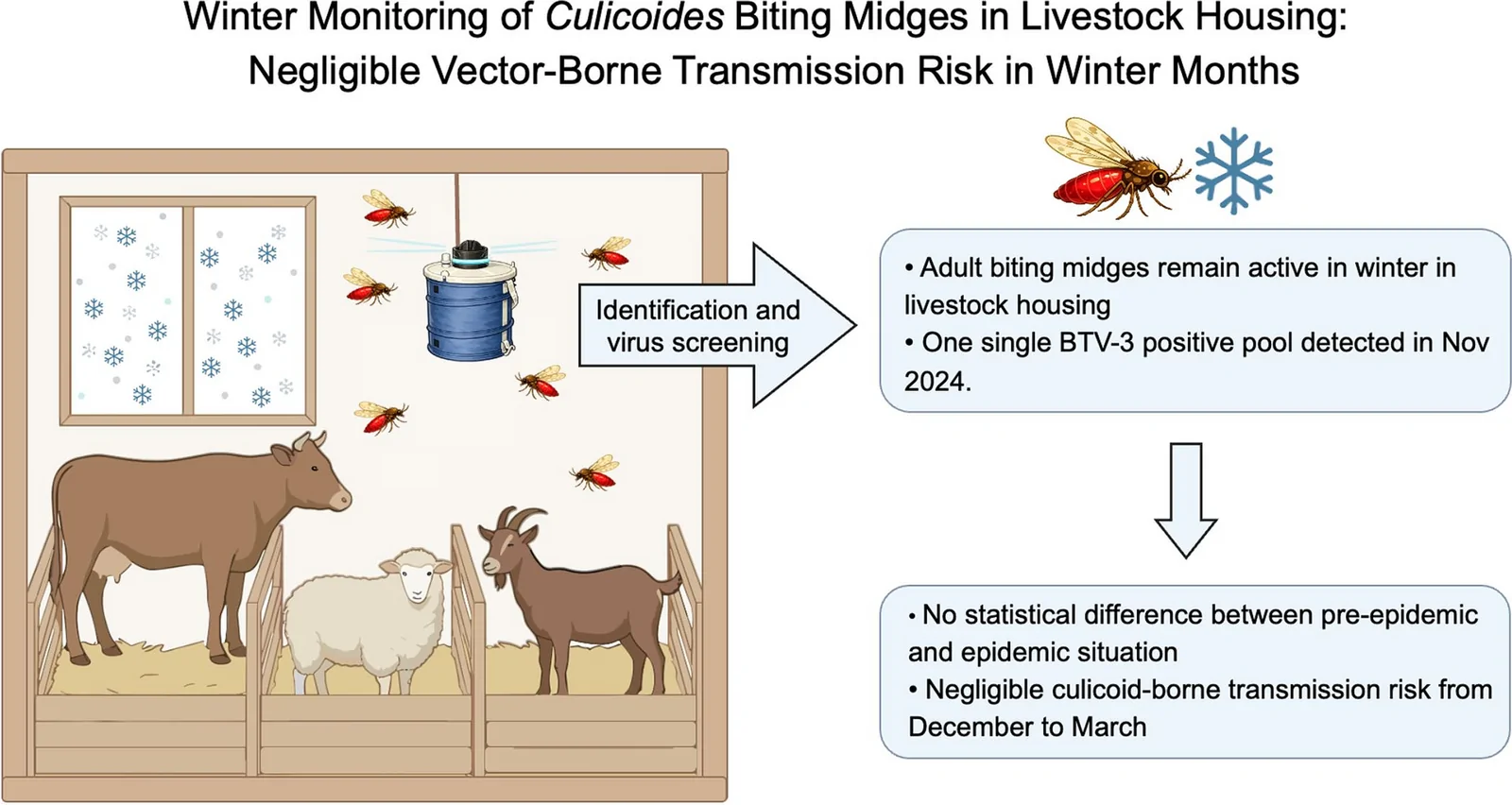

**Supplementary Information:**

The online version contains supplementary material available at https://doi.org/10.1186/s13071-026-07620-w.

## Background

Biting midges of the genus *Culicoides* Latreille 1809 (Diptera: Ceratopogonidae) are important vectors of arboviruses, with significant implications for veterinary health [1]. As important pathogens of ruminants, bluetongue virus (BTV; genus *Orbivirus*, family Sedoreoviridae), Schmallenberg virus (SBV; genus *Orthobunyavirus*, family Peribunyaviridae) and epizootic hemorrhagic disease virus (EHDV; genus *Orbivirus*, family Sedoreoviridae), are of particular concern in Europe. In a major part of Europe, the putative vectors of BTV and SBV are hematophagous females of member species of the Obsoletus Group and the Pulicaris Complex [1].

BTV primarily affects domestic and wild ruminants (e.g., sheep, cattle, goat, deer) as well as camelids, with infections ranging from subclinical to highly severe disease, including fever, weight loss, apathy, lameness, abortion and also death [2, 3]. It is a World Organisation for Animal Health (WOAH) notifiable disease, with its management regulated in the European Union under Regulation (EU) 2016/429 [4], supplemented by Regulation (EU) 2020/689 [5].

BTV serotype 8 (BTV-8) was introduced into central and northern Europe in 2006 and led to a multi-year epizootic with tremendous economic losses across numerous countries, including Germany [6], the Netherlands [7], and France [8]. The losses resulted from reduced productivity, increased veterinary interventions including vaccination, movement restrictions, international trade barriers, vector control measures and livestock mortality. In September 2023, BTV-3 was detected for the first time in continental Europe, beginning in the Netherlands [9] and subsequently spreading to Germany [10], the United Kingdom [11] and Belgium [12]. Until early September 2024, BTV-3 had also been detected in Luxembourg, France, Denmark and Switzerland [13]. By mid-December 2024, eight additional countries reported their first BTV-3 cases: Norway, Czechia, Austria, Sweden, Portugal, Spain, Greece and Poland [14–16]. By the end of October 2024, Germany alone had recorded nearly 13,200 BTV-3 outbreaks, with most of them reported in late summer 2024 (German Animal Disease Reporting System (TSN): www.tsn.fli.de), and livestock mortality in adult sheep in the Netherlands had amounted to 15.1% [17]. Since October 2025, BTV-8 has again been reported from Germany (new strain in the west, old strain in the east) with monthly new cases being notified throughout the winter until January 2026 (www.tsn.fli.de).

Unlike BTV, SBV has established an enzootic status in Germany since its first detection in 2011 [18] when it caused a major epidemic across Europe [19]. SBV infection during an early stage of gestation frequently results in abortion, stillbirths or severe malformations in calves and lambs [20–22].

A third *Culicoides*-borne arbovirus, EHDV, which is closely related to BTV, first emerged in Europe in November 2022 in Italy [23] but has since been reported from France [24], Spain [25] and Portugal [26]. In April 2025, a vaccinated cow imported from a high-risk area in France was found PCR-positive for EHDV in Belgium (Dierengezondheidszorg Vlaanderen, BE: www.dgz.be/nieuws/ehd-eerste-positief-geval-bij-dier-geimporteerd-uit-frankrijk). While EHDV affects white-tailed deer in North America [27], it is mainly a cattle pathogen and can also infect sheep subclinically. Clinical presentations in domestic ruminants are generally milder than for bluetongue, with cattle and white-tailed deer being the main reservoir species [25].

During the initial phase of the 2006 BTV outbreak in central and northern Europe, the vector–host transmission cycle had been expected to cease during wintertime and BTV to completely disappear again, as winter temperatures were assumed to be too low to sustain continued *Culicoides* activity and viral replication within the vector. However, recent evidence and a warming climate suggest that low-level viral transmission may persist year-round [28–30]. In fact, studies have documented evidence of overwintering and winter infections in Germany, for instance of SBV in sheep [30] and of BTV-8 in cattle [29], following the peaks of the epidemics. In both cases, infectious virus could be demonstrated (by inoculation of blood into mice, followed by seropositivity, and isolation on Vero cells) after detection via PCR. In the second case, the time of infection could be narrowed down to between January 20 to February 1, based on the time of seroconversion and becoming PCR-positive after being tested negative on January 22. These cases provide evidence that virus transmission may occasionally occur also in winter when climatic conditions are commonly considered unsuitable for vector activity [29]. According to recent surveillance data, 48 BTV-3 cases in ruminants were recorded for Germany from November 2023 to March 2024 and 3,628 cases during the subsequent winter of 2024/2025 (www.tsn.fli.dewww.tsn.fli.de), although—since cattle may remain PCR-positive for up to ca. 220 days following BTV-infection [31]—most of these are probably the results of infections during summer or autumn (see Discussion).

The mechanisms underlying activity and overwintering of both arboviruses and their *Culicoides* vectors remain unclear, but several hypotheses have been proposed, including transovarial transmission in the vector population, transplacental transmission in the ruminant population and transfer to the fetus, viral persistence in immune cell subsets of the ruminant hosts, and adult vector persistence [32, 33].

Adult *Culicoides*, including blood-fed individuals, have been recorded during winter months inside livestock housings in several parts of Europe, such as Germany [34–36] and Switzerland [37]. Under central Italian, Mediterranean climatic conditions, adults were trapped continuously throughout the colder season, both indoors and outdoors [38]. The EU concept of a ‘vector-free period’ during wintertime, describing critical reference criteria for suspending movement and trade restrictions on ruminants, thus demands re-evaluation. Current collection thresholds are defined by Regulation (EU) 2020/689 as less than five parous *Culicoides* females per trap and night and the complete absence of *C. imicola* Kieffer 1913 [5]. Although determination of parity needs experience and is characterized by moderate reliability [39], segregating parous from nulliparous *Culicoides* without specimen dissection is commonly performed just by visual inspection based on the absence or presence of a burgundy-red pigment inside the biting midge’s abdomen wall. This indicator of previous gravidity and follicle development is supposed to persist after oviposition [40]. However, winter counts of blood-fed *Culicoides* alone are insufficient to infer BTV circulation, which instead depends on the coupled dynamics of viremia, incident infections, and acquisition and onward transmission between holdings, distinct from general pathogen overwintering.

The primary objectives of the present study were therefore to assess both the winter activity of *Culicoides* biting midges and the number of blood-fed females per trap and night inside livestock housings across Germany as well as to check the biting midges for the occurrence of viral RNA as a proxy for viral presence and circulation in winter, before and during the BTV-3 outbreak. These data are to be assessed alongside biting midge longevity, duration of BTV viremia in ruminant hosts and the potential for low-temperature transmission between holdings to infer whether winter represents a period with active, ongoing virus circulation between vectors and hosts, or whether virus persistence depends on still unknown mechanisms independent of vector-mediated transmission.

## Methods

### Study area and sampling periods

The study was conducted during the four winter periods 2021/2022, 2022/2023, 2023/2024 and 2024/2025, from November 1 to March 31, respectively. Apart from the city-states Berlin, Bremen and Hamburg, monitored farms were distributed across all federal states of Germany.

In total, 80 unique locations with animal husbandry were monitored, including 12 farms in the first winter period, 34 in the second, 48 in the third and 55 in the fourth. Several farms participated in more than one season. The farms represented holdings of cattle (herd sizes ranging from 10 to 900 animals), sheep (5 to 400), goats (17 to 200) and horses (3 to 150). Housing and management types varied, with manure systems ranging from deep litter to concrete or slatted floor with scrapers, providing broad coverage of management practices. Cattle were typically housed indoors year-round, although some holdings offered access to adjacent pastures. By contrast, sheep and goats tended to be housed only at night, except for the lambing season. Horses were usually kept outdoors but had access to shelter or were occasionally stabled, depending on weather conditions and management practices.

### *Culicoides* collection and morphological identification

Biting midge collection was carried out with BG-Sentinel 2 UV-light traps (Biogents, Regensburg, Germany). Except for Marktredwitz (federal state of Bavaria, 2022/2023) and Sponholz (Mecklenburg-Western Pomerania, 2023/2024), which had three traps in three different livestock housings, and Marktredwitz (Bavaria, 2024/2025) and Schmergow (Brandenburg, 2024/2025), which had two traps in two different housings, all other farms had one trap installed in one livestock housing, despite some farms having multiple housings. Collections were carried out in more than one livestock housing on the same farm, if those varied considerably in construction, animal density or manure management, creating diverse (breeding habitat) conditions for biting midges.

The trap sites remained consistent throughout the study periods on farms that participated in more than one winter period. Across all four monitoring periods, 155 individual traps were deployed: 12 traps at 12 locations in winter 2021/2022, 36 traps at 34 locations in winter 2022/2023, 50 traps at 48 locations in winter 2023/2024 and 57 traps at 55 locations in winter 2024/2025.

Traps were activated once per week for 24 consecutive hours. Due to the BTV-3 outbreak, an increased sampling frequency was implemented in winter 2024/2025 at selected locations, where traps were operated multiple days per week (Additional file 2: Table S1). Sampling days were site-specific and not standardized across locations. All traps were operated indoors, thus protected from wind and rainfall, and positioned in close proximity to the livestock.

In the traps, insects were captured in a beaker filled with 80% ethanol in which they were stored at room temperature in the dark until further processing. All samples were examined under a stereomicroscope to separate *Culicoides* biting midges from by-catch and non-hematophagous Ceratopogonidae. Specimens belonging to the genus *Culicoides* were morphologically sorted using established identification keys [41, 42] and assigned to taxonomic groups based on observable characters. Due to morphological similarities and practical limitations in species-level identification, females were classified to Obsoletus Group (subgenus *Avaritia*), comprising *C. obsoletus* Meigen 1818, *C. scoticus* Downes and Kettle 1952, *C. chiopterus* Meigen 1818 plus *C. dewulfi* Goetghebuer 1936 [43]. The Pulicaris Complex (subgenus *Culicoides*) includes *C. pulicaris* Linnaeus 1758, *C. newsteadi* Austen 1921, *C. impunctatus* Goetghebuer 1920 and *C. punctatus* Meigen 1804 [44]. Blood-fed females were registered as separate categories of the group/complex.

The analysis of the collection data with respect to the EU Regulation 2020/689 [5] to determine a vector-free period was conducted based on blood-fed females instead of parous ones. Although parity determination through abdominal pigmentation provides some indication of age structure, the inherent uncertainties in distinguishing parous from nulliparous specimens precluded its use as a reliable criterion. Since blood-fed females will become parous and may include parous ones, the ratio of parous to nulliparous females should be much higher than that of blood-fed to non-blood-fed females. Using blood-fed females as a proxy is therefore conservative, as it underestimates the total population of potentially parous females. This approach provides a lower-bound estimate of vector competence status within collections. The number of parous females and females to become parous within collections is therefore always higher than the number of blood-fed females.

### Climate data collection

UV-light traps were equipped with data loggers (EBI 20-THI; Xylem Analytics, Ingolstadt, Germany) to record temperature at hourly intervals. However, due to technical and operational issues, continuous temperature data were not produced for all trapping sites. Analyzable temperature records were obtained from 8 of 12 sites in the 2021/2022 season, from all 36 sites in 2022/2023, from 33 of 50 sites in 2023/2024 and from 55 of 57 sites in 2024/2025.

### Virus detection in *Culicoides*

According to collection date and location, all Obsoletus Group and Pulicaris Complex female biting midges of the three winter monitoring periods 2022/2023, 2023/2024 and 2024/2025 were sorted into pools of up to 50 individuals. *Culicoides* collected during winter 2021/2022 were not included in the virological analyses, as this period preceded the BTV-3 outbreak in Germany by almost two years and was therefore not in the focus of analytical efforts on periods of high epidemiological relevance. For viral screening, the biting midges were dried to remove residual ethanol, homogenized and tested with multiplex quantitative reverse transcription PCRs (RT-qPCR) targeting BTV- and EHDV-RNA as described by Wernike et al. [45] and SBV-RNA as described by Bilk et al. [46]. Pools positive for viral RNA were meant to be subsequently analyzed genetically for included *Culicoides* species using the identification assays developed by Dähn et al. [47, 48].

### Data analysis

Data processing was conducted in R (4.5.1; R Core Team) with customized scripts. Geospatial data handling was performed in QGIS (3.40.5 ‘Bratislava’; QGIS Development Team). Projected coordinate systems were applied using ETRS89/LCC Germany (N-E) (EPSG:4839). Calendar week (cw) assignment followed DIN 1355-1 (ISO 8601), meaning that a single cw may include days from two different consecutive months and, thus, does not necessarily align with the calendar month.

## Results

### *Culicoides* collection and taxonomic assignment

Weekly UV-light trap catches of Obsoletus Group and/or Pulicaris Complex varied both spatially and temporally across the four analyzed winter periods. Notably, *Culicoides* activity during the study period was mostly limited to November, December and March with only sporadic catches in January and February. Throughout the four sampling periods, 2,822 trap collections were conducted (186 in 2021/2022; 558 in 2022/2023; 704 in 2023/2024; 1,374 in 2024/2025), resulting in the morphological identification of 50,898 female *Culicoides* specimens belonging to the Obsoletus Group and Pulicaris Complex (Table 1). Of these, the Obsoletus Group comprised 87.8% (n = 44,681) and the Pulicaris Complex 12.2% (n = 6,217). Most of the collected *Culicoides* females were non-blood-fed (98.9%, n = 50,320), while 1.1% (n = 578) were blood-fed, consisting of 87.5% (n = 506) Obsoletus Group and 12.5% (n = 72) Pulicaris Complex. A total of 4,540 Obsoletus Group and Pulicaris Complex individuals were collected during the first winter period (2021/2022; Additional file 3: Table S2), 965 during the second period (2022/2023; Additional file 4: Table S3), 7,918 during the third period (2023/2024; Additional file 5: Table S4) and 37,475 during the fourth period (2024/2025; Additional file 6: Table S5).

**Table 1:** Numbers of collected Obsoletus Group and Pulicaris Complex females according to month of the respective sampling period and blood-feeding status

Winter	Month	Total	Obsoletus Group			Pulicaris Complex		
			Total	Non-blood-fed	Blood-fed	Total	Non-blood-fed	Blood-fed
2021/2022	Nov	4,070	3,900	3,872	28	170	164	6
	Dec	135	133	118	15	2	2	0
	Jan	19	19	19	0	0	0	0
	Feb	0	0	0	0	0	0	0
	Mar	316	315	315	0	1	1	0
	Subtotal	4,540	4,367	4,324	43	173	167	6
2022/2023	Nov	729	650	625	25	79	79	0
	Dec	27	27	27	0	0	0	0
	Jan	47	47	47	0	0	0	0
	Feb	2	2	2	0	0	0	0
	Mar	160	160	160	0	0	0	0
	Subtotal	965	886	861	25	79	79	0
2023/2024	Nov	1,901	1,844	1,771	73	57	57	0
	Dec	160	157	150	7	3	3	0
	Jan	98	94	94	0	4	4	0
	Feb	7	7	7	0	0	0	0
	Mar	5,752	4,836	4,671	165	916	852	64
	Subtotal	7,818	6,938	6,693	245	980	916	64
2024/2025	Nov	5,016	3,494	3,474	20	1,522	1,522	0
	Dec	1,516	1,470	1,462	8	46	46	0
	Jan	109	98	98	0	11	11	0
	Feb	360	356	356	0	4	4	0
	Mar	30,474	27,072	26,907	165	3,402	3,400	2
	Subtotal	37,475	32,490	32,297	193	4,985	4,983	2
Total		50,798	44,681	44,175	506	6,217	6,145	72

### Monthly activity patterns of female *Culicoides*

**Fig. 1:**
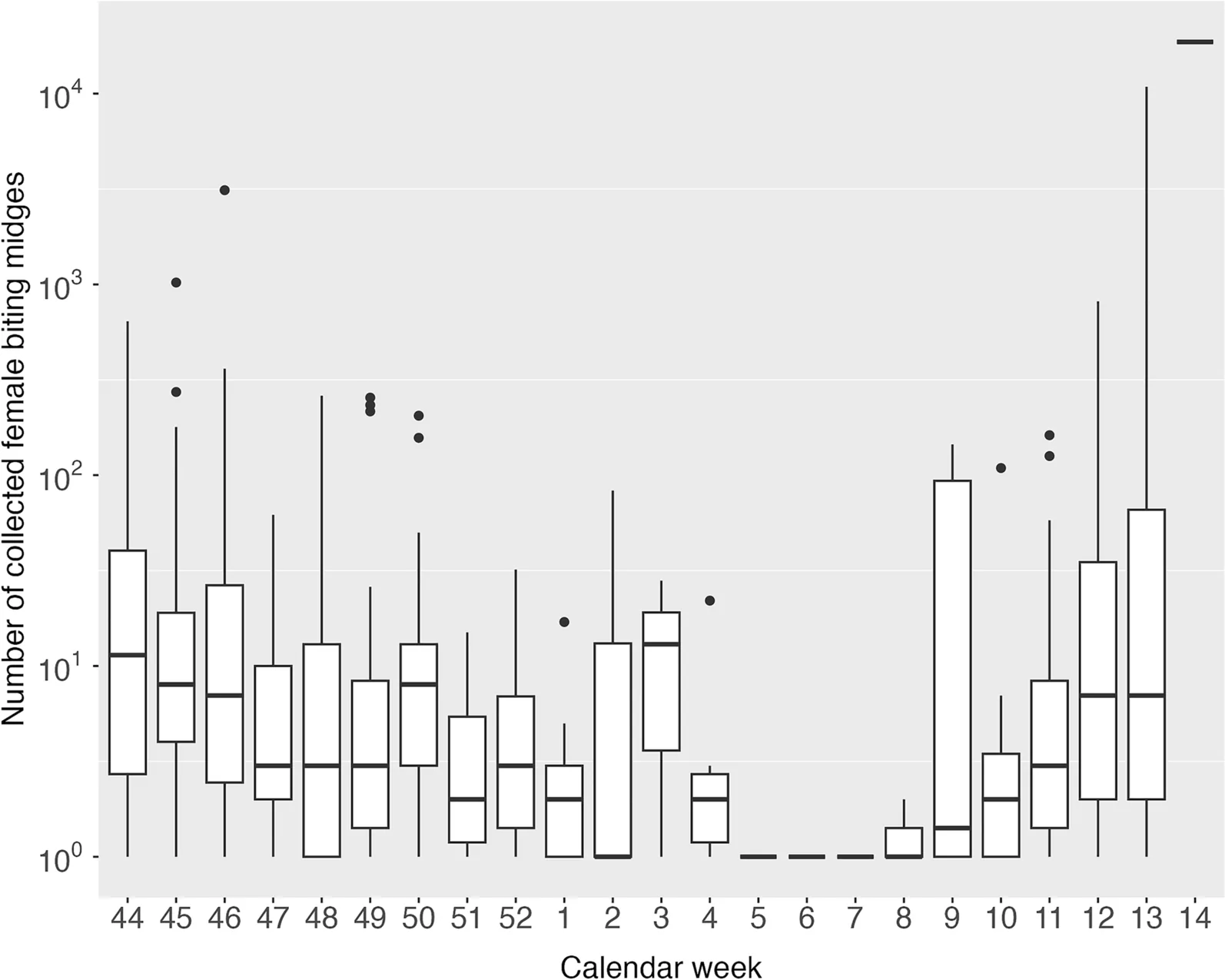
Numbers of Obsoletus Group and Pulicaris Complex females captured from November 1 to March 31 during four consecutive winter periods: 2021/2022, 2022/2023, 2023/2024 and 2024/2025. Boxplots indicate the median (line) and interquartile range (box); whiskers extend to 1.5xIQR (Tukey definition), with values beyond shown as outliers

Across all winter phases and sampling sites, observed numbers of Obsoletus Group and/or Pulicaris Complex females could be divided visually into three temporal phases: a ‘phase of declining activity’, a ‘phase of minimal activity’ and a ‘phase of increasing activity’ (Fig. 1). Between cw 44 of the previous year and cw 4 of the following year (roughly early November to mid/late January), both the median values and the interquartile ranges declined steadily. During this phase, catches declined by several orders of magnitude, although some cws deviated from this general trend. With respect to median values and variability, overall trapping numbers were lowest between cw 4 and cw 8, corresponding to mid/late January and mid/late February. From cw 9 onwards, a pronounced increase in catches was observed, marking the onset of a phase of steadily rising activity that continued until the end of March, which corresponded with the end of the studied periods.

Female collection numbers broken down to Obsoletus Group and Pulicaris Complex varied markedly over the four winter periods (Table 1). However, to provide an overview of general monthly patterns across years, the following numbers represent total counts, summed for each month over all four monitoring periods. For the Obsoletus Group females, captures declined from 9,888 female individuals in November to 1,787 in December and 258 in January. Since 9 individuals were collected in February 2022 to 2024, the decline continued, while—against the trend—356 females were collected in February 2025. Of these, 354 individuals were captured during three consecutive 24 h sampling days at a single site, indicating an effect of increased sampling frequency. The general decline was followed by a sharp increase to 32,383 individuals in March. A similar pattern was observed for the Pulicaris Complex females, with a total of 1,828 specimens collected in November, 51 in December, 15 in January, 4 in February and 4,319 in March.

**Fig. 2:**
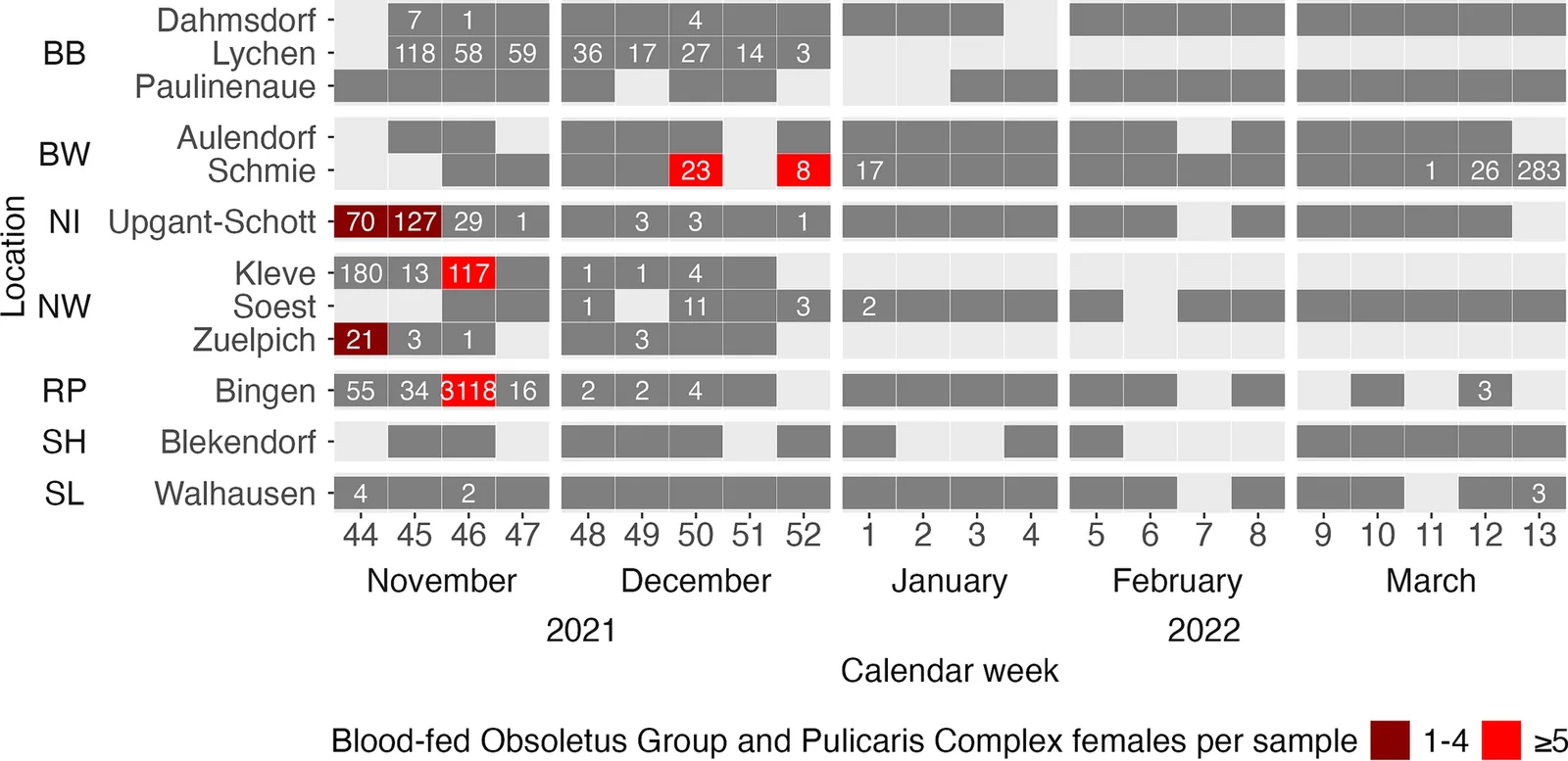
Overview of collected samples and Obsoletus Group and Pulicaris Complex females captured within 24 hours per calendar week from November 2021 to March 2022, using an individual UV-light trap. Each horizontal line represents the sampling record of one trap/site. Light grey indicates no sample, dark grey without a number denotes a sample without Obsoletus Group or Pulicaris Complex females, dark grey with number indicates the number of Obsoletus Group and Pulicaris Complex females captured. Dark red highlights samples with 1–4 blood-fed individuals and signal red highlights samples with ≥ 5 individuals. BB, Federal state of Brandenburg; BW, Baden-Wuerttemberg; NI, Lower Saxony; NW, North Rhine-Westphalia; RP, Rhineland Palatinate; SH, Schleswig-Holstein; SL, Saarland

**Fig. 3:**
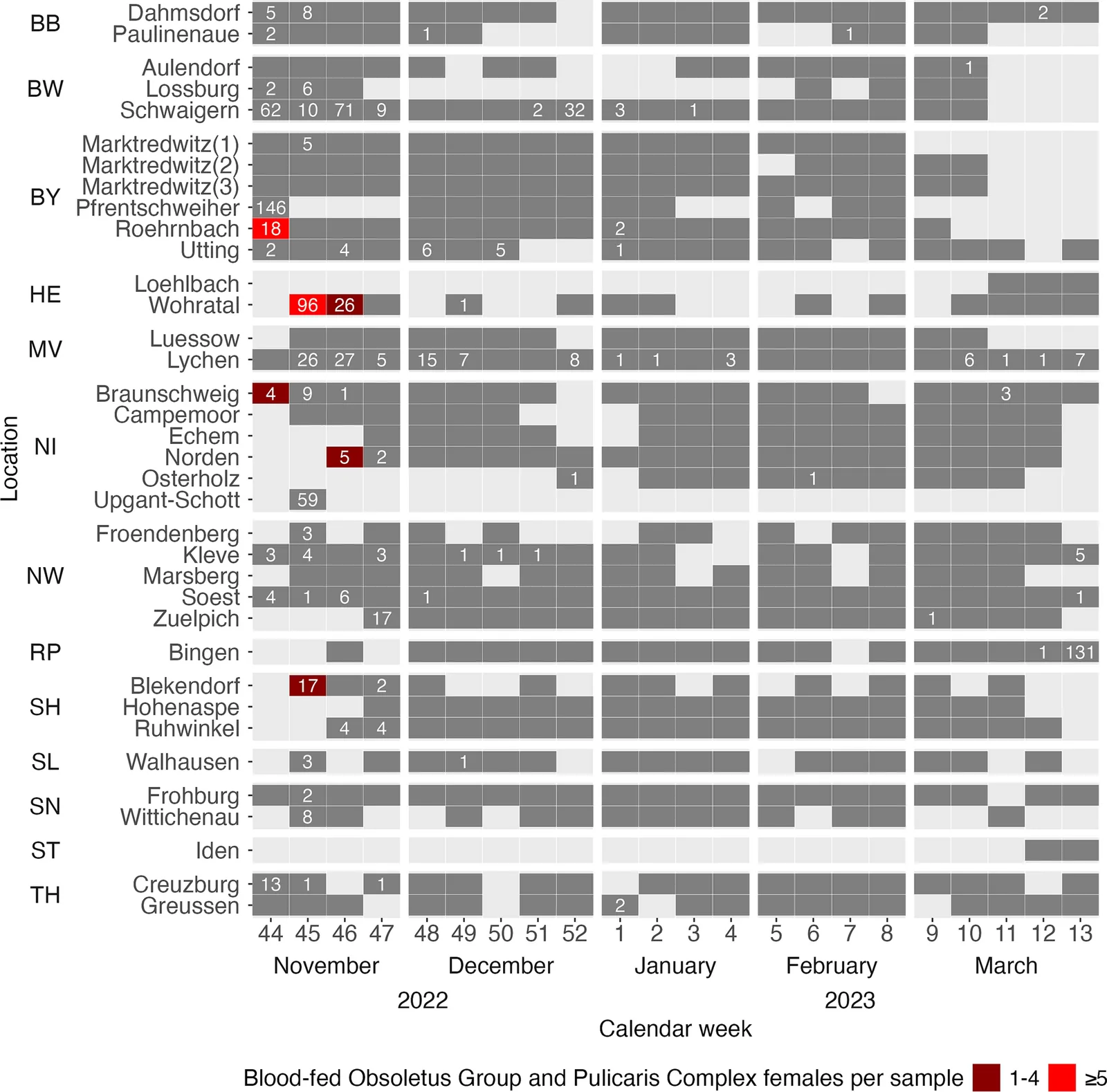
Overview of collected samples and Obsoletus Group and Pulicaris Complex females captured within 24 hours per calendar week from November 2022 to March 2023, using an individual UV-light trap. Each horizontal line represents the sampling record of one trap. Light grey indicates no sample, dark grey without a number denotes a sample without Obsoletus Group or Pulicaris Complex females, dark grey with number indicates the number of Obsoletus Group and Pulicaris Complex females captured. Dark red highlights samples with 1–4 blood-fed individuals and signal red highlights samples with ≥ 5 individuals. BB, Federal state of Brandenburg; BW, Baden-Wuerttemberg; BY, Bavaria; HE, Hesse; MV, Mecklenburg-Western Pomerania; NI, Lower Saxony; NW, North Rhine-Westphalia; RP, Rhineland-Palatinate; SH, Schleswig-Holstein

**Fig. 4:**
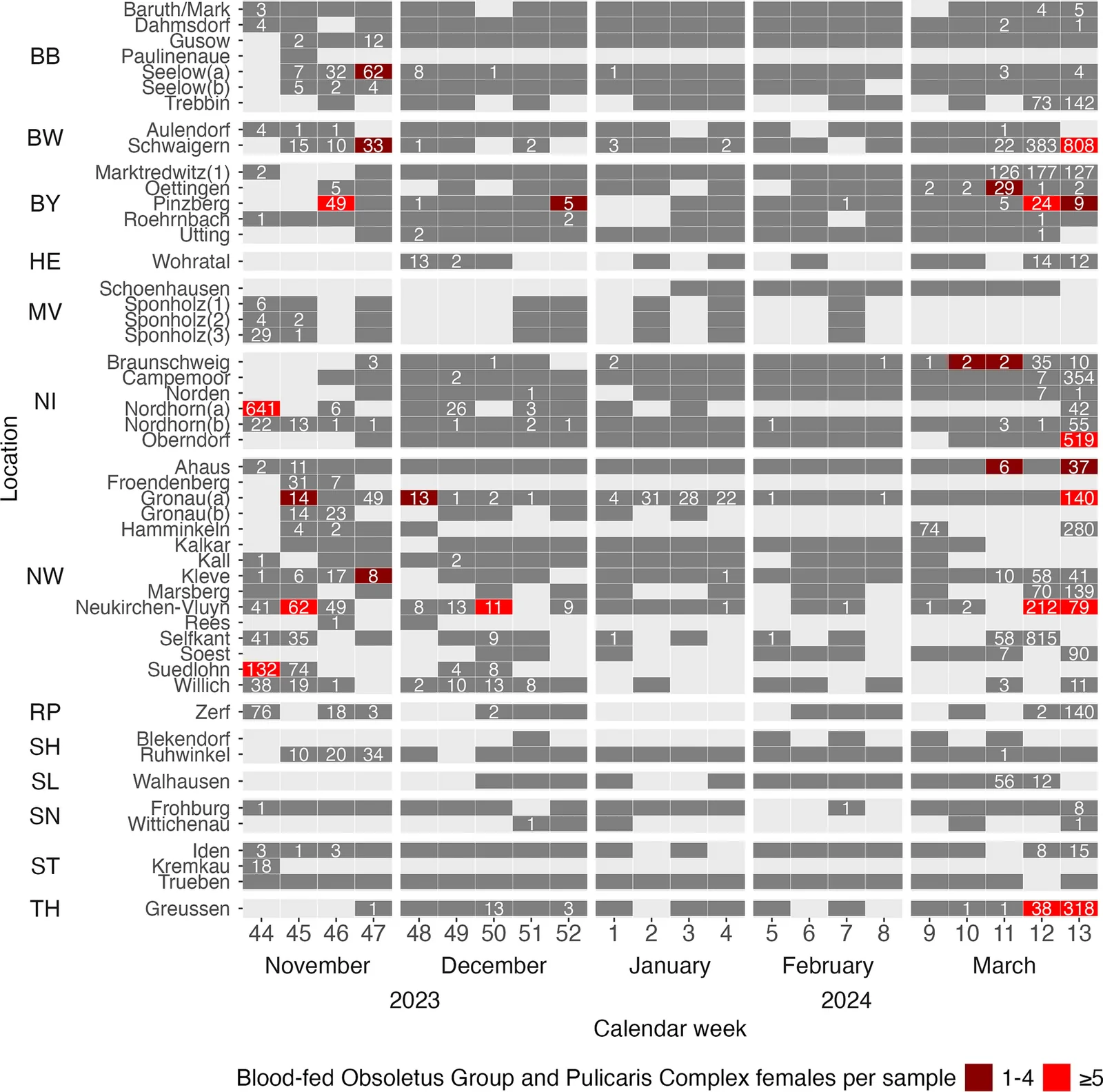
Overview of collected samples and Obsoletus Group and Pulicaris Complex females captured within 24 hours per calendar week, from November 2023 to March 2024, using an individual UV-light trap. Each horizontal line represents the sampling record of one trap. Light grey indicates no sample; dark grey without a number denotes a sample without Obsoletus Group or Pulicaris Complex females; dark grey with number indicates the number of Obsoletus Group and Pulicaris Complex females captured. Dark red highlights samples with 1–4 blood-fed individuals and signal red highlights samples with ≥ 5 individuals. BB, Federal state of Brandenburg; BW, Baden-Wuerttemberg; BY, Bavaria; HE, Hesse; MV, Mecklenburg-Western Pomerania; NI, Lower Saxony; NW, North Rhine-Westphalia; RP, Rhineland-Palatinate; SH, Schleswig-Holstein; SL, Saarland; SN, Saxony; ST, Saxony-Anhalt; TH, Thuringia

**Fig. 5:**
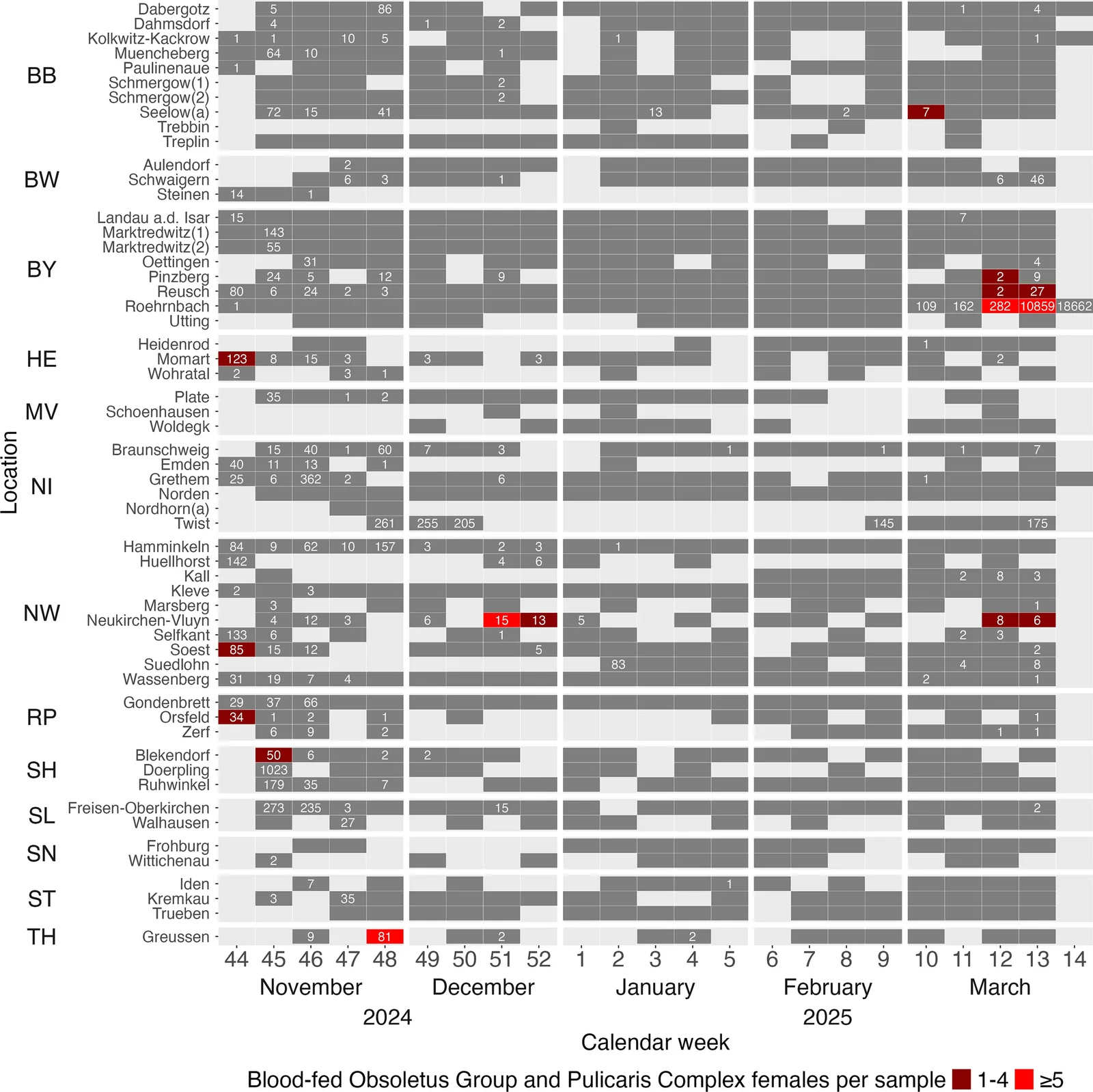
Overview of collected samples and Obsoletus Group and Pulicaris Complex females captured within 24 hours per calendar week, from November 2024 to March 2025, using an individual UV-light trap. For weeks with multiple 24 hour-trap collections at a given site, the weekly value was defined as the 24 hour-catch yielding the highest number of captured female biting midges. Each horizontal line represents the sampling record of one trap. Light grey indicates no sample; dark grey without a number denotes a sample without Obsoletus Group or Pulicaris Complex females; dark grey with number indicates the number of Obsoletus Group and Pulicaris Complex females captured. Dark red highlights samples with 1–4 blood-fed individuals and signal red highlights samples with ≥ 5 individuals. BB, Federal state of Brandenburg; BW, Baden-Wuerttemberg; BY, Bavaria; HE, Hesse; MV, Mecklenburg-Western Pomerania; NI, Lower Saxony; NW, North Rhine-Westphalia; RP, Rhineland-Palatinate; SH, Schleswig-Holstein; SL, Saarland; SN, Saxony; ST, Saxony-Anhalt; TH, Thuringia

The numbers of collected Obsoletus Group and/or Pulicaris Complex females per location, trapping week and winter period are visualized as heatmaps (Figs. 2-5), allowing a direct comparison of catch intensity across calendar weeks and sites.

### Collection of blood-fed *Culicoides* females

**Fig. 6:**
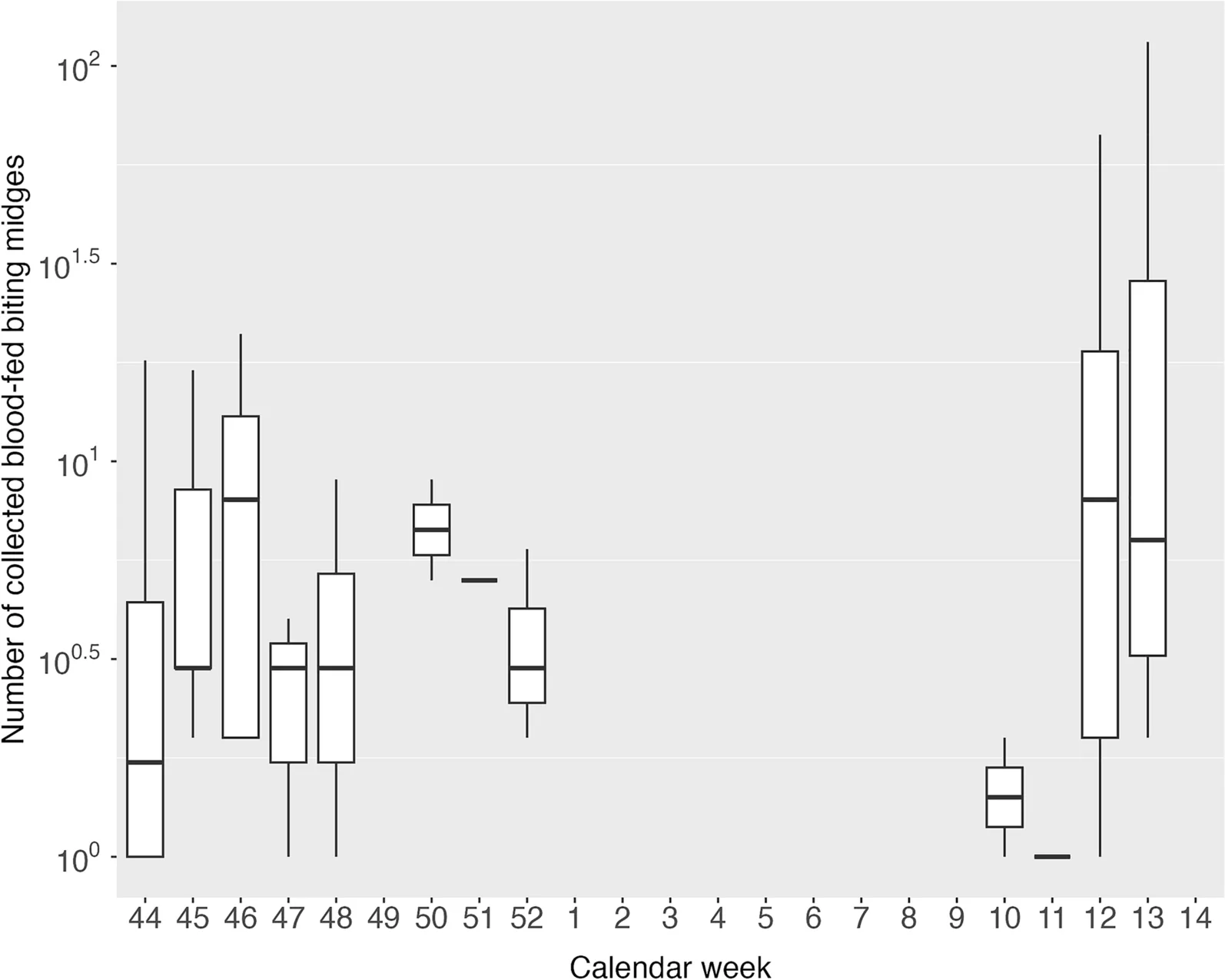
Numbers of blood-fed Obsoletus Group and Pulicaris Complex females captured from November 1 to March 31 during four consecutive winter periods: 2021/2022, 2022/2023, 2023/2024 and 2024/2025. Boxplots indicate the median (line) and interquartile range (box); whiskers extend to 1.5xIQR (Tukey definition), with values beyond shown as outliers

Across all analyzed winter seasons and the different locations, weekly catches of blood-fed Obsoletus Group and/or Pulicaris Complex followed a temporal pattern that could be visually divided into three phases (Fig. 6). Phase 1, spanning cw 44 to cw 52 (early November to late December), was characterized by the presence of blood-fed females in every week, albeit at very low absolute numbers. During this phase, the indoor catches showed pronounced variability, reflected by wide interquartile ranges and therefore substantial differences between weeks and sites. Phase 2, covering cw 1 to cw 9 of the following year (early January to late February/early March), was marked by the complete absence of blood-fed females across all winters and all locations. In phase 3 (cw 10–14; early to late March), blood-fed females were detected again. This renewed increase in biting-midge abundance was accompanied by high variability, with wide interquartile ranges indicating pronounced differences between locations.

In the four monitoring periods, a total of 578 blood-fed Obsoletus Group and Pulicaris Complex females was captured. In the first winter period (2021/2022), all 43 blood-fed Obsoletus Group and six Pulicaris Complex females (Table 1) were collected in November and December at five different locations (Additional file 3: Table S2). At the beginning of November (cws 44–45), up to four blood-fed females per 24 h were recorded at two sites each, while at three other locations at least five blood-fed females each were captured per 24 h in November and December (cws 46, 50, 52) (Fig. 2). In the second winter period (2022/2023), all 25 blood-fed individuals belonged exclusively to the Obsoletus Group (Table 1) and were trapped in November (cws 44 to 46) at five different locations (Additional file 4: Table S3). At three sites, up to four blood-fed females each were collected per 24 h, and at two sites at least five blood-females each were captured per 24 h (Fig.3).

In contrast to the previous winter seasons, captures of blood-fed females were more frequent in the monitoring winter 2023/2024 (Fig. 4): a total of 245 blood-fed Obsoletus Group and 64 Pulicaris Complex (Table 1) females were collected in November, December and March at 13 locations (Additional file 5: Table S4). During that winter period, numerous sites across the majority of the federal states showed blood-fed females, beginning in November and extending into December, with detections again occurring in March (cws 10–13) at about one third of the monitored locations. During the fourth winter period 2024/2025 (Fig. 5), a total of 193 blood-fed females of the Obsoletus Group and two of the Pulicaris Complex (Table 1) were captured in November, December and March across ten locations (Additional file 6: Table S5) with four samples exceeding the threshold of >4 blood-fed females at three locations.

Notably, across all monitored winter seasons, none of the January or February samples contained blood-fed *Culicoides*. The proportion of blood-fed females relative to total catch remained consistent across winter months, ranging from 0–2% (November 1%, December 2%, January 0%, February 0%, March 1%). Most samples with at least one blood-fed female were collected per 24 h during cws 44 to 48, with numbers showing a continuous decline from early November to early December, remaining low throughout December and increasing again from cws 10 to 13 in March (Additional file 1: Fig. S1).

**Fig. 7:**
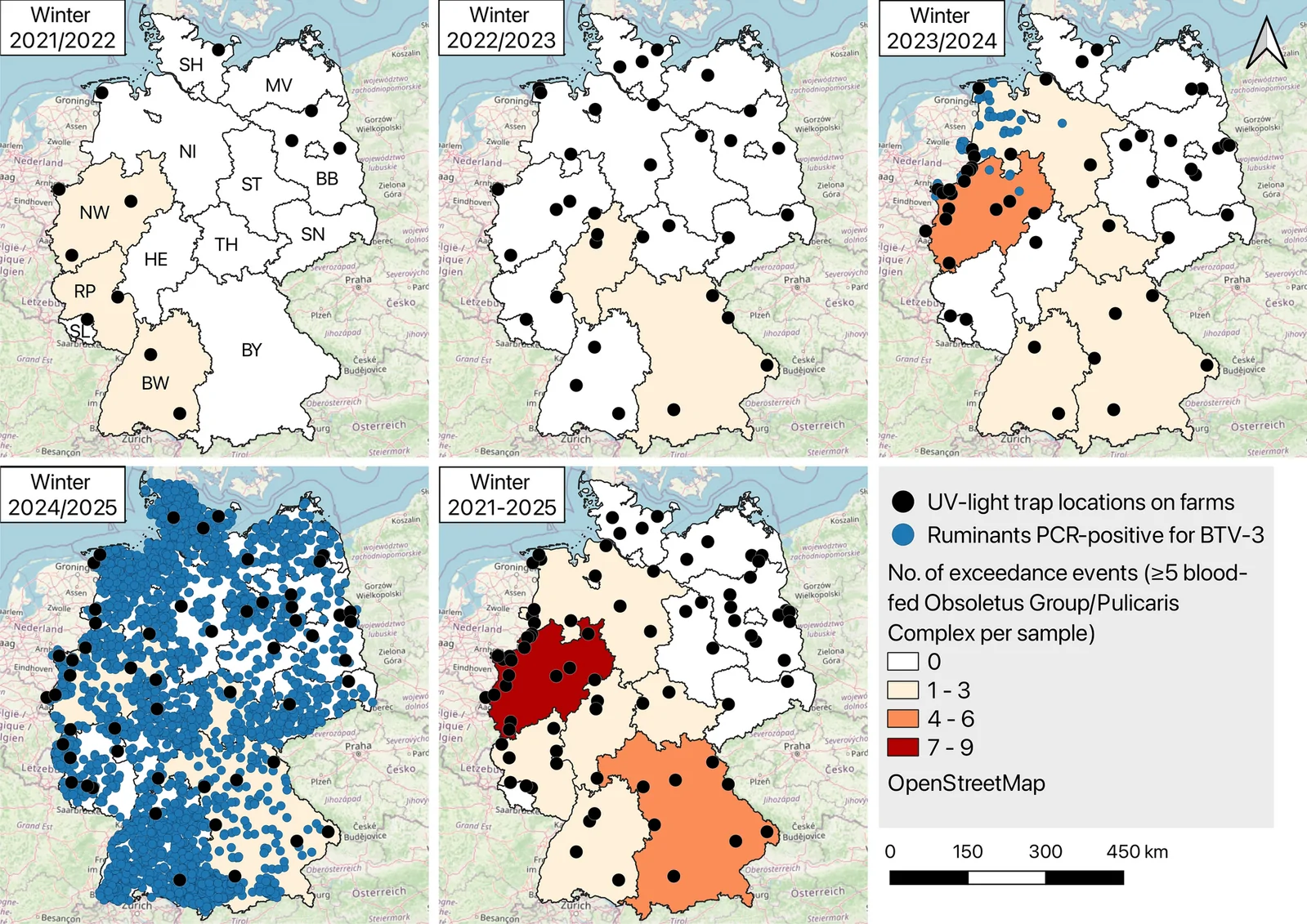
Winter monitoring of *Culicoides* in Germany from 2021/2022 to 2024/2025. Maps show UV-light trap locations on farms (black dots), PCR confirmed BTV-3 infections in ruminants (blue dots; source: TSN) and the number of exceedance events (≥ 5 blood-fed Obsoletus Group/Pulicaris Complex females per sample) per federal state in the respective winter period (color gradient). Panels represent individual winters (November-March) and the cumulative period 2021–2025 (bottom right). BB, Federal state of Brandenburg; BW, Baden-Wuerttemberg; BY, Bavaria; HE, Hesse; MV, Mecklenburg-Western Pomerania; NI, Lower Saxony; NW, North Rhine-Westphalia; RP, Rhineland-Palatinate; SH, Schleswig-Holstein; SL, Saarland; SN, Saxony; ST, Saxony-Anhalt; TH, Thuringia

The spatial distribution of trapping locations with more than four captured blood-fed Obsoletus Group/Pulicaris Complex females per 24 h varied markedly during the study periods (Fig. 7). During the first two winter periods (2021/2022 and 2022/2023), only a few events exceeding the threshold were detected at sites in southern and western Germany. During the 2023/2024 monitoring period, clusters of increased *Culicoides* blood-feeding activity appeared mainly in western Germany and, to a lesser extent, in the south and northwest. In the subsequent winter (2024/2025), exceedance events where again low and confined to southern and western federal states, whereas BTV-3-PCR-positive ruminants were reported from all federal states. When data from all monitoring periods (2021–2025) were aggregated, the highest frequencies of exceedance events were observed in western and southern Germany with a distinct hotspot in the federal state of North Rhine-Westphalia, followed by Bavaria.

### Virus detection

Across all winter collections from 2022/2023 (n = 131 pools, 965 females), 2023/2024 (n = 375 pools, 7,918 females), and 2024/2025 (n = 1,075 pools, 37,475 females) tested for BTV, SBV, and EHDV, only a single pool was positive for BTV-3 RNA (Ct 36.5); all remaining pools were PCR-negative. The single positive pool consisted of 50 Obsoletus Group females collected on November 1, 2024 at the cattle farm Huellhorst (North Rhine-Westphalia). Subsequent genetic analysis identified the presence of *C. obsoletus* clade O1, *C. scoticus* and *C. dewulfi* in that pool.

### Temperature variations across winter periods

Mean monthly air temperatures at the trapping sites varied between 3.6 °C and 11.9 °C (Table 2). November and March were consistently the warmest months, whereas December and January represented the coldest months in all years. The mildest winter was recorded in 2023/2024 with an overall mean temperature of 8.1°C, followed by 2021/2022 and 2022/2023 (7.5 °C each), while 2024/2025 was the coldest study period with an average of 6.5 °C. Across winters, November exhibited the highest mean temperature (10.5 °C) and January the lowest (4.8 °C).

**Table 2:** Monthly mean air temperatures [°C] for each winter monitoring period, including averages across winters and months

Month	2021/2022	2022/2023	2023/2024	2024/2025	Mean across winters
Nov	11.86	9.92	9.61	10.63	10.50
Dec	5.93	5.60	6.66	6.17	6.09
Jan	4.87	6.42	4.31	3.57	4.79
Feb	6.76	6.64	9.05	3.78	6.56
Mar	7.85	8.89	10.66	8.47	8.96
Mean across months	7.45	7.49	8.06	6.52	/

**Fig. 8:**
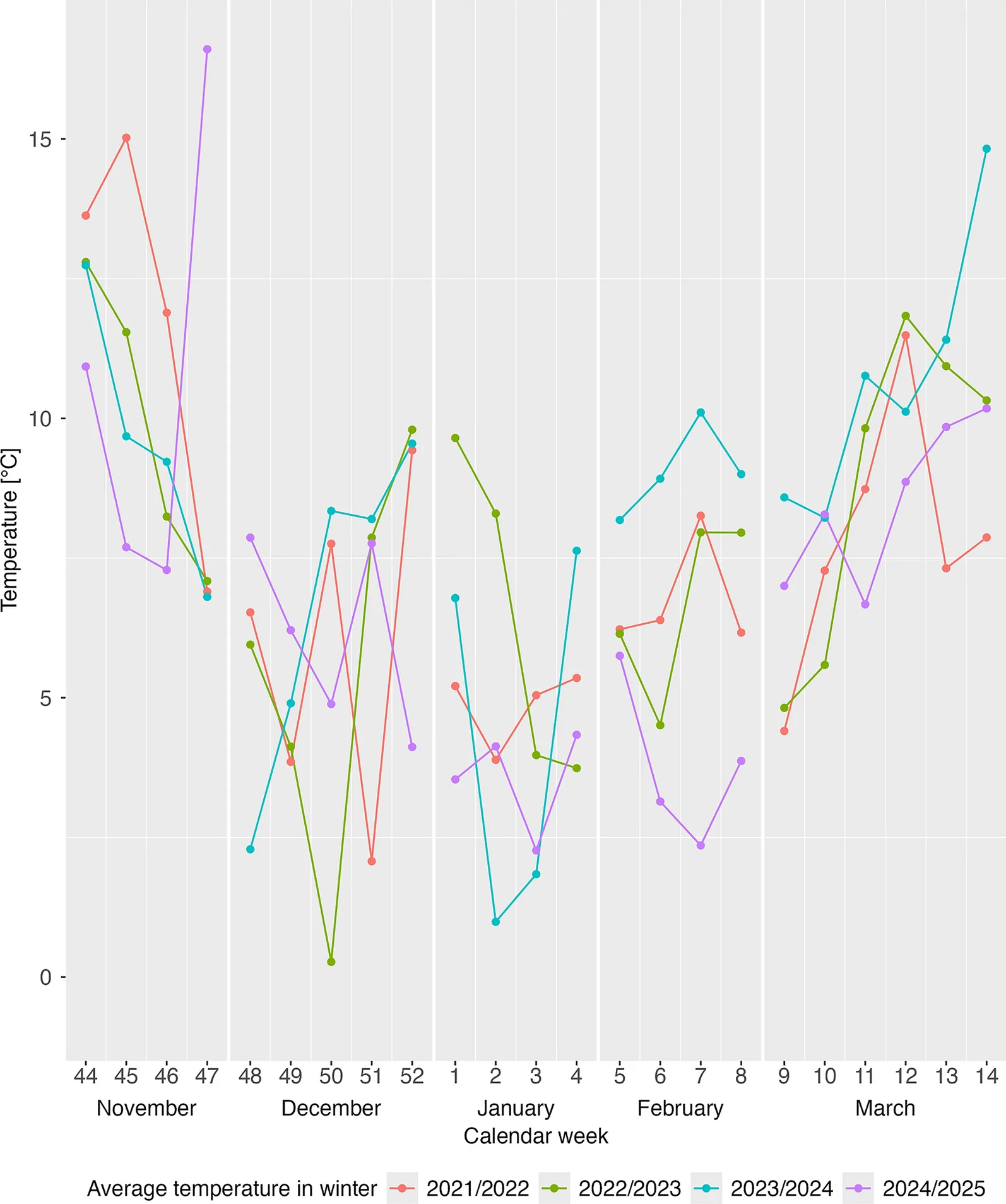
Average weekly temperatures during the winter periods 2021/2022 (red), 2022/2023 (green), 2023/2024 (blue) and 2024/2025 (purple) according to calendar week

Average weekly temperatures showed distinct interannual variation in the four winter periods (Fig. 8, Additional file 7: Table S6). In November, temperatures generally started between 10.9 °C and 13.8 °C and decreased progressively into December. In the winter of 2021/2022, the lowest weekly average temperature was recorded in the penultimate week of December (2.0 °C), whereas in 2022/2023 this was the case already in mid-December (0.2 °C), in 2023/2024 as early as in the beginning of December (2.5 °C) and in 2024/2025 in the penultimate week of January (2.2 °C). Despite these differences, temperatures during the final week of December were remarkably similar across the first three study seasons (9.4–9.8 °C on average), whereas in the fourth monitored season they averaged to 4.1 °C. While average weekly temperatures in February increased slightly, except for 2024/2025, the most pronounced temperature peak occurred in March of the 2023/2024 winter period (average of 10.7 °C). The end of the observation periods in March 2022/2023 and March 2024/2025 were characterized by temperatures around 11 °C, in contrast to 7.3 °C in March 2021/2022.

## Discussion

The present study provides a most detailed, multi-season, Germany-wide assessment of winter activity and virus infection status of *Culicoides* biting midges of the Obsoletus Group and Pulicaris Complex in livestock housing under temperate central European conditions. This is particularly important given the emergence and re-emergence of highly relevant biting midge-borne viruses in Europe, such as BTV, SBV and EHDV, and the ongoing question of whether these viruses can persist, replicate, and be transmitted during central European winters. The study covers the periods before and during the central European BTV-3 epidemic and, thus, facilitates a unique comparison of pre-outbreak and outbreak conditions regarding virus circulation. It therefore enables evidence-based conclusions to inform wintertime restriction measures and related regulations.

UV-light trap monitoring revealed the presence of potential arbovirus vectors inside stables throughout winter, with variations between sites, years and local climatic conditions. Overall, female *Culicoides* activity declined from November to February and increased again in March, consistent with previous studies from German livestock environments [36, 49–51]. February consistently exhibited the lowest adult *Culicoides* activity in this study, corresponding to other studies, where February was the month with the lowest or among the months with the lowest activity levels [35, 36, 49]. The complete absence of Obsoletus Group and Pulicaris Complex biting midges in February 2022 highlights interannual variability but does not alter the general pattern.

Marked variability in winter activity of *Culicoides* between sites suggests a strong influence of local factors such as farm management practices, housing conditions and microclimate. While some locations showed little or no activity throughout an entire winter season, others consistently recorded low-level presence, emphasizing the need for multi-year and multi-site entomological surveillance and site-specific analysis of potential influencing factors.

Across all monitored winter periods, the Obsoletus Group dominated the collections, indicating a stable species composition and corroborating previous findings from livestock housings (e.g., [34, 52–54]). In addition to their overall higher abundance in central Europe [55], winter activity and ongoing development of Obsoletus Group members in stables, as compared to the outdoor situation, are made possible not only by sufficiently high temperatures but also by the provision of larval habitats. Manure-enriched organic substrates, feed residues and composted plant material commonly found in livestock environments have been identified as key breeding habitats for this group [56], with deep litter bedding recently been confirmed [57]. By contrast, species of the Pulicaris Complex are rather associated with a range of (naturally) moist microhabitats not present in stables [56, 58], which likely explains their low indoor numbers during winter. Additionally, the endophilic tendency of Obsoletus Group species enhances their abundance in livestock housing, whereas the exophilic behavior of Pulicaris Complex species contributes to their low indoor abundance [59].

Blood-fed females were mainly detected in November and March, with only sporadic records in December and none in January and February, consistent with observations from previous German winter studies [36]. Analysis of blood-fed proportions revealed that feeding rates remained relatively consistent across winter months (1–2%), indicating that the absence of blood-fed females in January and February reflected low overall vector abundance rather than suppressed feeding activity. This pattern suggests that biting midge females continue to emerge and exhibit host-seeking behavior in stables into early winter, then resume in late winter as soon as environmental conditions improve, while outdoor activity remains largely suppressed by unfavorable weather [35, 36, 49]. However, short-term *Culicoides* activity peaks during mild winter days followed by a rapid decline in activity once temperatures drop again, have also been described [38, 60, 61]. The pronounced increase in blood-fed specimens in March 2024 coincided with unusually mild temperatures and was not reproduced in the following winter, indicating a climatic rather than site-driven effect.

Previous latitude-based analyses have demonstrated that the onset of the vector season occurs at progressively lower mean temperatures towards northern Europe [55]. While vector activity in southern latitude ranges (Spain and southern France) was reported to begin at mean temperatures of approx. 10–12 °C, vector season onset at northern latitudes (Scandinavian countries) occurred at mean temperatures as low as 1–3 °C. This pattern suggests a climatic adaptation of *Culicoides* populations to cooler environments [55]. However, temperature thresholds for vector activity (indoor and outdoor) and for virus replication must be considered separately, as vector activity can occur at temperatures well below those required for virus replication within the vector [62].

Unlike certain mosquito species, which overwinter as adult females and may survive several months in a state of dormancy [63], *Culicoides* are not known to hibernate as long-lived dormant adults. Under natural winter conditions, biting midges are assumed to overwinter predominantly as fourth-instar larvae, with adults completely absent [64]. During that time, *Culicoides* development is generally suppressed due to subthreshold developmental conditions, allowing population build-up not before spring. The indoor detection of adult *Culicoides* during winter months therefore implies ongoing development under appropriate conditions, most likely attributable to above threshold temperatures in relatively warm livestock housing and breeding substrates (e.g., deep litter, dungheaps). Two mechanisms may explain the persistence of adult *Culicoides* during winter: first, continued emergence of new individuals. Stokes et al. [65] showed that Obsoletus Group specimens can continue to emerge, albeit at very low rates, even during winter months when suitable developmental conditions persist. Second, extended survival of adult *Culicoides* (longevity) at cold temperatures, typical of many insects under low-temperature conditions. Laboratory studies have demonstrated that adult *Culicoides* exhibit specific thermal preferences [66], which they likely exploit within livestock facilities to locate optimal microhabitats, potentially balancing metabolic demands with thermal shelter.

Although the detection of adult *Culicoides* during winter months provides evidence for local activity, vector presence alone is insufficient to infer virus transmission. For virus transmission risk assessments, the distinction between parous and nulliparous *Culicoides* is central. Having completed at least one gonotrophic cycle after a blood meal, parous females might be infectious during following bites, provided the first blood host was infectious as well and passed on the virus to the biting midge. By contrast, nulliparous individuals are considered non-infectious since they have never had the chance to infect themselves on a vertebrate host. An exception to the latter would be the situation of vertical transmission, which could render females infectious with emergence, i.e., before the first blood meal. While transovarial transmission pathways have been suggested for SBV [67] and EHDV [68], there is up to now no evidence for transovarial transmission of BTV. The practical assessment of parity in field-collected biting midges is, however, methodologically constrained. Morphological parity classification based on abdominal pigmentation is prone to misdiagnosis, with false parity rates of up to 23% [39, 40] and incomplete pigmentation in some parous females [69]. While this limits non-destructive field diagnostics, viral RNA testing of females, though more resource-intensive, would yield more reliable information on vector status. Furthermore, for risk assessment, it is particularly important to note that the bite of a single infected biting midge is sufficient to infect a ruminant [70], whereas only a small fraction of biting midges (0–7.3%) feeding on a viremic mammalian host becomes infected [71]. Field studies have reported minimum infection rates (= number of infected vectors/total vectors examined × 1,000) ranging from 135.47 to 333.13 under high virus circulation conditions [10, 72] and from 3.75 to 72.45 under low virus circulation conditions [72].

Against this background, virus detection in *Culicoides* trapped inside stables during the winter season was extremely rare in this study: only one single, BTV-3 PCR-positive pool was identified among the 1,581 pools tested. This pool, collected on November 1, 2024 on a cattle farm in North Rhine-Westphalia, consisted of Obsoletus Group females, and genetic analysis confirmed the presence of *C. obsoletus* clade O1, *C. scoticus* and *C. dewulfi*. These species are among those discussed by Uiterwijk et al. [73] as potential central European BTV-3 vectors, based on PCR detection in field-collected biting midges in combination with ecological factors relevant to vector capacity, such as host association, seasonal abundance and population size [74]. The PCR result was associated with a relatively high Ct-value, indicating a low viral RNA load in the sample which may reflect early or declining infections. Previous studies have reported the detection of SBV in *Culicoides* pools collected in November [72, 75] while other investigations testing *Culicoides* captured in November for BTV [76] or SBV [77] exclusively reported negative results. With the exception of Zeiske et al. [72], no study extended sampling and testing beyond November. The present study therefore is the first to assess virus presence in biting midge pools collected throughout the winter season. The detection of the positive biting midge pool in this study occurred during a period that coincided with the large epidemic wave of BTV-3 that spread across Germany and affected all federal states. During that outbreak, 3,628 ruminants (bovines: 93.05%, n = 3,376; small ruminants: 4.33%, n = 157; wild cervids and caprids: 2.07%, n = 75; camelids: 0.55%, n = 20; TSN: www.tsn.fli.de) tested PCR-positive for BTV-3 between November 2024 and March 2025 (November: 1,064; December: 722; January: 906; February: 597; March: 339). Contrasting this, 48 BTV-3 PCR-positive ruminants (bovines: 97.92%, n = 47; small ruminants: 2.08%, n = 1) had been reported between November 2023 and March 2024 (November: 10; December: 9; January: 13; February: 7; March: 9; www.tsn.fli.de), following the onset of the BTV-3 outbreak in Germany in October 2023. In the winter 2023/2024, as in the winter before the BTV-3-outbreak, none of the tested *Culicoides*-pools turned out positive for BTV-3-RNA. Strikingly, the increased virus abundance in the host population during the winter 2024/2025 was not reflected by a higher winter prevalence of PCR-positive biting midge pools. This apparent discrepancy can be explained by several factors: first, BTV infection in ruminants is characterized by long-term PCR detectability (up to ca. 220 days) of viral RNA, caused by the persistence of non-infectious subviral particles, including RNA, associated with erythrocytes [31, 78]. Based on experimental and field data, the WOAH assumes a viremic, infectious period of approximately 60 days for BTV-infected ruminants, with a probability of > 99% that detectable viremia in adult cattle resolves before week 9 post-infection [79]. Accordingly, PCR-based surveillance massively overestimates the duration of infectious virus circulation in ruminant populations. Given the late summer and early autumn peak in blood-fed *Culicoides* and SBV-RNA positive biting midge pools [10, 72], livestock infections detected in winter therefore most likely originate from the preceding period of vector activity. In mosquitoes, it has been shown that host-seeking behavior intensifies towards the end of the major annual activity period, driven by physiological changes and environmental factors such as declining temperatures [80]. A similar seasonal mechanism may also apply to biting midges, potentially enhancing their host-seeking activity in late summer and early autumn and thereby increasing the likelihood of virus transmission to ruminant hosts.

Second, virus replication in *Culicoides* requires sustained temperatures of at least 11–13 °C [62] which substantially exceed the temperature thresholds above which biting midges may be active in wintertime [55, 81]. According to the weekly average temperatures across monitoring sites per period, such temperatures were rarely reached during the study periods. Only in November and March did a few cws at some sites show weekly average temperatures above this threshold. Consequently, host viremia in winter generally becomes epidemiologically irrelevant for further transmission, as virus circulation is primarily limited by temperature-dependent extrinsic virus replication and the availability of viraemic hosts. This is further corroborated by the observation that for SBV and BTV, although shown to circulate at considerable levels among biting midges during the summer and autumn seasons 2022 and 2024, respectively (Zeiske et al., unpublished data), no corresponding increase in vector infection prevalences was detected in the subsequent winters. Notwithstanding, occasional reports of BTV and SBV infections in ruminants during the winter season [29, 30] indicate that sporadic transmission or long-term survival of infected biting midges under cold-season conditions may occur. However, such isolated cases do not suggest relevant or ongoing virus circulation. Although biting midges might be responsible for very rare transmission events within the same barn building provided indoor temperatures allow for virus replication, they do not seem to be active outside livestock housings during wintertime [35, 36, 82], preventing a potential transfer of virus from one farm to another. However, even low-level transmission could represent an epidemiologically relevant overwintering mechanism, as a newly infected ruminant would maintain infectious viremia until spring, enabling broader virus circulation once vector populations recover and temperatures permit general viral replication. This possibility, however, remains speculative given the principal absence of virus-positive *Culicoides* in our extensive monitoring during an outbreak scenario.

If BTV-infections occurred in ruminants in late autumn, the virus could theoretically persist throughout the winter via interconnected transmission chains. Considering a life span of adult biting midges of about three weeks or—at low winter temperatures—maybe longer, indoor blood feeding, and a viremia of 60 days in infected ruminants, the virus could well be brought through the winter by ongoing transmission between biting midge and ruminant, provided stable temperatures allow completion of the viral EIP.

A comparison with the lowest MIR reported in the literature for low-level virus circulation in Germany (3.75 [72]) provides context. The MIR conservatively assumes a minimum of one infected individual per positive pool and thus provides a standardized estimate of individual infection probability. Applying a MIR of 3.75, approx. 174 infected biting midge females would have been expected among the 46,358 tested in the present study (46,358 × 3.75/1,000 = 173.8). Based on the observed average pool size (29.32; 46,358/1,581), this would correspond to approx. 164 positive pools under a binomial assumption (probability ≥ 1 infected individual per pool: 1-(1–0.00375)^29.32 = 0.104; 0.104 × 1,581 = 164). By contrast, only a single positive pool was detected.

The numeric threshold of Regulation (EU) 2020/689 [5] of less than five parous female *Culicoides* collected per trap and night to suspend trade restrictions for ruminants can be traced back to Meiswinkel et al. [83]. The authors explained that the former criterion of < 10 *Culicoides* per night (based on observations reported by Losson et al. [84] from Belgium) required refinement, as it did not account for age-grading. Since only a fraction of trapped females would be parous (with an even smaller fraction potentially BTV-infected), the criterion was strengthened by introducing the amendment ‘< 5 parous *Culicoides*’. This recommendation was based on the finding that > 99% of the < 100 *Culicoides* collected between January and March 2007 in the Netherlands were nulliparous and thus represented no transmission risk, even when catches occasionally exceeded 10 individuals per trap and night. Meiswinkel et al. [83] attributed these data to unpublished observations by Meiswinkel and Dijkstra from 2007. Moreover, there is currently no evidence for the presence of *C. imicola* in central Europe [85], including Germany, which is consistent with previous entomological surveys [50, 51]. To date, the northernmost confirmed finding of established *C. imicola* populations in Europe originates from the Var department in southern France [86]. Apparently, the detection of a single *C. imicola* specimen in southern Switzerland appeared to represent a wind-dispersed vagrant from more southern populations [87].

## Conclusions

Taken together, the limited evidence of BTV-3 circulation in *Culicoides* biting midges captured during the winter months of 2024/2025 is consistent with the current knowledge on the temporally restricted duration of viremia (and, thus, infectiousness) in ruminants and the strong temperature dependence of virus replication within the insect vector. Although epidemiologically relevant *Culicoides* were present during the winter months, vector presence alone is insufficient to infer virus transmission. Infectious host viremia is temporally limited to approx. 60 days while extrinsic virus replication in biting midges is largely inhibited under winter temperature conditions in Germany. Furthermore, increased PCR positivity in ruminants during winter does not necessarily reflect ongoing, epidemiologically relevant virus circulation. In addition, regulatory concepts or risk assessments that rely on the presence or absence of *C. imicola* are not applicable to the epidemiological situation in Germany, where biting midge-borne virus transmission is primarily driven by *Culicoides* species of the Obsoletus Group. According to the above argumentation, the concept of a ‘vector-free period’ may benefit from a refinement. Rather than ‘vector-free’, which implies complete absence of vectors, alternative terminology such as ‘vector-low period’ (https://www.gov.uk/government/news/seasonally-vector-low-period-for-bluetongue-begins) may provide a biologically more meaningful framework. This aligns with Napp et al. [88], who argue that complete cessation of vector activity is restricted to Afrotropical species in southern Europe, while in temperate Europe a ‘period of low vector activity’ better describes conditions under which transmission risk is low rather than absent. However, we propose that the term ‘replication-free period’ would emphasize the temperature-dependent inhibition of virus replication in vectors, even when adult *Culicoides* (including blood-fed females) are detected.

Blood-feeding activity by biting midges at the end of December was documented in the present study, and—although no blood-fed biting midges were caught in January and February, cases of fresh infections in ruminants during these months had been documented by Hoffmann et al. [29] and Wernike et al. [30]. In spring, when *Culicoides* activity increases and populations grow, a single viraemic host could catalyze rapid amplification and transmission to numerous vectors, thereby initiating the seasonal outbreak cycle. The key question remains: how do biting midges infected during the period December–February find the temperatures required for virus replication when ambient temperatures are assumed to be generally lower than those needed for EIP completion? It is conceivable that sufficiently high temperatures could be maintained through a combination of thermal preference behavior of the biting midges and microclimatic conditions within livestock housing (e.g., deep litter bedding or other local thermal microhabitats). As we found only a single positive biting midge pool in the large-scale, multi-site and multi-year surveillance approach presented here at the start of winter, when the BTV-3 outbreak in Germany reached its peak, the described, interconnected transmission chain is likely to occur only in very rare cases. It remains unclear whether such isolated cases are sufficient to explain the persistence of the virus in winter.

Based on the presented data, the risk of virus transmission by biting midges is considered negligible in Germany from December to March, as winter temperatures are generally insufficient to permit sustained extrinsic virus replication in *Culicoides*, infectious host viremia is temporally limited, and outdoor vector activity is largely absent. Under current climate change trajectories, a continued decline in the frequency of cold spells and frost days is expected in central Europe [89]. This trend could have direct temporal and spatial implications for ‘replication-free periods’, although most likely not in the near future.

## Supplementary Information

Supplementary Material 1. **Figure S1.** Proportion of samples per calendar week containing at least one blood-fed *Culicoides* female of the Obsoletus Group or Pulicaris Complex across all monitoring periods. Bars represent the relative frequency of samples with (red) and without (grey) blood-fed individuals.

Supplementary Material 2. **Table S1.** Number of samples per location and calendar week during the respective winter period (2021/2022, 2022/2023, 2023/2024, 2024/2025).

Supplementary Material 3. **Table S2.** Trapping numbers and virus testing results of Obsoletus Group and Pulicaris Complex in winter period 2021/2022.

Supplementary Material 4. **Table S3.** Trapping numbers and virus testing results of Obsoletus Group and Pulicaris Complex in winter period 2022/2023.

Supplementary Material 5. **Table S4.** Trapping numbers and virus testing results of Obsoletus Group and Pulicaris Complex in winter period 2023/2024.

Supplementary Material 6. **Table S5.** Trapping numbers and virus testing results of Obsoletus Group and Pulicaris Complex in winter period 2024/2025.

Supplementary Material 7. **Table S6.** Weekly average temperatures for each calendar week in the winter periods 2021–2025.

## Data Availability

Data are provided within the manuscript or supplementary information files.

## Abbreviations

BTV: Bluetongue virus
SBV: Schmallenberg virus
EHDV: Epizootic hemorrhagic disease virus
PCR: Polymerase chain reaction
RT: Reverse transcriptase
TSN: Tierseuchen-Nachrichtensystem (German Animal Disease Reporting System)
